# Effect of polymerised type I collagen on hyperinflammation of adult outpatients with symptomatic COVID‐19

**DOI:** 10.1002/ctm2.763

**Published:** 2022-03-16

**Authors:** Silvia Méndez‐Flores, Ángel Priego‐Ranero, Daniel Azamar‐Llamas, Héctor Olvera‐Prado, Kenia Ilian Rivas‐Redonda, Eric Ochoa‐Hein, Andric Perez‐Ortiz, Mario E. Rendón‐Macías, Estefano Rojas‐Castañeda, Said Urbina‐Terán, Luis Septién‐Stute, Thierry Hernández‐Gilsoul, Adrián Andrés Aguilar‐Morgan, Dheni A. Fernández‐Camargo, Elizabeth Olivares‐Martínez, Diego F. Hernández‐Ramírez, Gonzalo Torres‐Villalobos, Janette Furuzawa‐Carballeda

**Affiliations:** ^1^ Department of Dermatology Instituto Nacional de Ciencias Médicas y Nutrición Salvador Zubirán Mexico City Mexico; ^2^ Department of Internal Medicine Instituto Nacional de Ciencias Médicas y Nutrición Salvador Zubirán Mexico City Mexico; ^3^ Department of Anesthesiology Instituto Nacional de Ciencias Médicas y Nutrición Salvador Zubirán Mexico City Mexico; ^4^ Department of Immunology and Rheumatology Instituto Nacional de Ciencias Médicas y Nutrición Salvador Zubirán Mexico City Mexico; ^5^ Department of Epidemiology Instituto Nacional de Ciencias Médicas y Nutrición Salvador Zubirán Mexico City Mexico; ^6^ Escuela de Medicina Ciudad de México Mexico City, Mexico Universidad Panamericana; ^7^ Division of Surgery, Massachusetts General Hospital Boston MA USA; ^8^ Emergency Department Instituto Nacional de Ciencias Médicas y Nutrición Salvador Zubirán Mexico City Mexico; ^9^ Department of Pneumology Instituto Nacional de Ciencias Médicas y Nutrición Salvador Zubirán Mexico City Mexico; ^10^ Facultad de Medicina Mexico City, Mexico Universidad Nacional Autónoma de México; ^11^ Department of Nephrology and Mineral Metabolism Instituto Nacional de Ciencias Médicas y Nutrición Salvador Zubirán Mexico City Mexico; ^12^ Departments of Experimental Surgery and Surgery Instituto Nacional de Ciencias Médicas y Nutrición Salvador Zubirán Mexico City Mexico

Dear Editor,

Although dexamethasone is approved for the hyperinflammation treatment of hospitalised COVID‐19 patients, non‐hospitalised patients do not benefit from this therapy.^1^ A potential drug for treating COVID‐19 patients is polymerised type I collagen (PTIC). A downregulator of pro‐inflammatory cytokines, adhesion molecules (ELAM‐1, VCAM‐1, and ICAM‐1), cyclooxygenase (Cox)‐1 enzyme and the collagenases expression through the modulation of transcription of factor NF‐kB.^2^, ^3^, ^4^, ^5^, ^6^ The intramuscular or subcutaneous administration of PTIC to patients with active RA (Phase II studies) improved the count of swollen joints and morning stiffness; 57% of patients achieved an ACR score of 50, and 30% had disease remission with this therapeutic combination. PTIC was safe and well‐tolerated in long‐term treatment, without adverse effects.^7^, ^8^, ^9^

A double‐blind, randomised, placebo‐controlled clinical trial evaluated the PTIC intramuscular administration's safety and efficacy on hyperinflammation, oxygen saturation and symptom improvement in adult symptomatic COVID‐19 outpatients (https://www.medrxiv.org/content/10.1101/2021.05.12.21257133v1).

Eighty‐nine participants with a confirmed COVID‐19 diagnosis (mild to moderate disease) were included from August 31 to November 7, 2020, and followed for 12 weeks. Patients were randomly assigned to receive either 1.5 ml of PTIC intramuscularly every 12 h for 3 days and then every 24 h for 4 days (*n* = 45) or a matching placebo (*n* = 44) (sample size is describe in Methodology S1). Demographics, clinical characteristics, coexisting conditions and symptoms are described in Table 1. Ninety‐eight per cent of patients in the PTIC group and 95.5% in the placebo group were analysed by the intention‐to‐treat principle (Figure S1). Of 89 patients at baseline, 64 (72%) were being treated with acetaminophen, 28 (31.5%) with acetylsalicylic acid, 5 (5.6%) with antivirals and 36 (40.4%) with antibiotics. The use of acetaminophen (71% vs. 73%), acetylsalicylic acid (27% vs. 39%), antivirals (7% vs. 5%) and antibiotics (40% vs. 41%) were similar in the PTIC and placebo groups, respectively. No patients were treated with anticoagulants or steroids.

**TABLE 1:** Baseline demographic and clinical characteristics of the trial population

Characteristic	All subjects (*N* = 89)	PTCI (*N* = 45)	Placebo (*N* = 44)	*p* Value
**Comparability of randomised groups**				
Age (years), mean ± SD Median Range	48.5 ± 14.1 48.0 19.0–78.0	48.4 ± 14.4 47.0 19.0–77.0	48.6 ± 13.9 48.0 22.0–78.0	.9917
18–39 years, *n* (%)	24 (27.0)	13 (28.9)	11 (25.0)	.7585
40–64 years, *n* (%)	52 (58.4)	25 (55.6)	27 (61.4)	
65+ years, *n* (%)	13 (14.6)	7 (16.3)	6 (13.6)	
Male sex, *n* (%)	37 (41.6)	18 (40.0)	19 (44.2)	.9008
BMI (kg/m^2^), mean ±SD Median Range	28.0 ± 4.5 27.9 18.6–40.8	27.8 ± 4.5 27.9 18.6–40.3	28.2 ± 4.5 27.7 20.1–40.8	.7934
Overweight, *n* (%)	39 (43.8)	21 (46.7)	18 (40.1)	.3847
Obesity, *n* (%)	25 (28.1)	11 (25.0)	14 (32.5)	.4758
Baseline Guangzhou Severity Index, mean ± SD Median Range	87.6 ± 25.9 90.1 29.4–137.5	87.9 ± 30.2 92.0 29.4–135.1	87.3 ± 20.8 88.7 35.5–137.5	.4362
Baseline Chest CT Score <20% ≥20% 20–50% >50%	53 (59.6) 20 (22.5)	27 (60.0) 8 (17.8) 5 (11.1) 3 (6.7)	26 (59.1) 12 (27.3) 12 (27.3) 0 (0.0)	.3353
Days from symptom onset to onset of treatment (Median, IQR)	7.0 (4.0)	7.0 (4.0)	7.0 (4.0)	.7257
Oxygen Saturation				
pSO2 ≤ 92% (%)	28 (31.5)	13 (28.5)	16 (36.4)	.325
pSO2; mean ± SD Median IQR	92 ± 2.5 92.0 –91 to 94	93 ± 2.0 93 –91 to 95	92 ± 2.9 92 –91 to 93	.252
**Laboratory variables**				
Complete blood count				
Leukocyte count (×103/μl), mean ± SD Median Range	5.87 ± 2.08 5.30 2.80–12.50	6.03 ± 2.04 5.60 2.80–12.40	5.70 ± 2.13 5.00 3.00–12.50	.240^b^
Haemoglobin (g/dl), mean ± SD Median Range	15.48 ± 1.72 15.30 10.50–20.10	15.50 ± 1.80 15.40 11.90–20.10	15.45 ± 1.66 15.15 10.50–18.70	.743^a^
Platelets (K/μl), mean ± SD Median Range	273.80 ± 116.16 249 73–910	283.18 ± 130.35 249 148–910	264.20 ± 100.21 250 73–568	.625^b^
Lymphocyte count (%), mean ± SD Median Range	30.13 ± 10.79 30.80 8–57	30.15 ± 10.99 31.40 8.1–57	30.13 ± 10.72 30.45 8–54	0.866^a^
Neutrophil count (%), mean ± SD Median Range	60.05 ± 11.23 58.70 31–82	59.89 ± 11.82 58.70 31–81	60.22 ± 10.73 58.85 39–82	.835^a^
Neutrophil‐lymphocyte ratio (NLR), mean ± SD Median Range	2.58 ± 1.91 1.88 0.54–10.25	2.62 ± 2.05 1.81 0.54–9.93	2.53 ± 1.78 1.91 0.72–10.25	.931^b^
Liver function test (LFT)				
Total bilirubin (mg/dl), mean ± SD Median Range	0.62 ± 0.28 0.56 0.18–1.87	0.62 ± 0.24 0.54 0.26–1.34	0.62 ± 0.33 0.57 0.18–1.87	.709^b^
Direct bilirubin (mg/dl), mean ± SD Median Range	0.13 ± 0.07 0.11 0.03–0.44	0.13 ± 0.06 0.11 0.04–0.33	0.14 ± 0.08 0.12 0.03–0.44	.372^b^
Indirect bilirubin (mg/dl), mean ± SD Median Range	0.49 ± 0.22 0.45 0.15–1.56	0.49 ± 0.19 0.45 0.22–1.11	0.49 ± 0.26 0.46 0.15–1.56	.617^b^
Aminotransferase, serum aspartate (AST) (U/L), mean ± SD Median Range	31.09 ± 20.82 26 9–158	28.39 ± 15.60 22 11–83	33.87 ± 24.97 27.50 9 –1 58	.150^b^
Aminotransferase, serum alanine (ALT) (U/L), mean ± SD Median Range	37.42 ± 28.14 29.80 7–129.80	35.64 ±29.90 23 9–129.80	39.24 ± 26.43 31.50 7–120	.176^b^
Albumin (g/dl), mean ± SD Median Range	4.35 ± 0.44 4.34 2.55–5.71	4.40 ± 0.50 4.43 2.55–5.71	4.32 ± 0.38 4.30 3.52–5.45	.189^b^
Fasting glucose (mg/dl) Mean ± SD Median Range	116.75 ± 61.85 98 66–386	119.31 ± 64.32 102 66–386	114.14 ± 59.86 96.50 72–354	.380^b^
Lactate dehydrogenase (LDH) (U/L) Mean ± SD Median Range	166.70 ± 50.59 155 97–325	165.09 ± 60.76 150 97–325	168.34 ± 38.15 160 99–311	.500^b^
C‐reactive protein (high sensitivity) (mg/dl) Mean ± SD Median Range	1.63 ± 2.58 0.73 0.02–16.47	1.32 ± 2.67 0.50 0.05–16.47	1.95 ± 2.49 0.97 0.02–11.49	.650^b^
Ferritin (ng/ml) Mean ± SD Median Range	243.46 ± 285.20 161.70 4–1614.40	235.14 ± 293.70 161.70 4–1614.40	251.96 ± 279.39 161.45 5.60–1277	.599^b^
D‐dimer (ng/dl) Mean ± SD Median Range	1106.74 ± 3537.99 456 185–29948	1732.33 ± 4916.88 491 185–29948	466.93 ± 225.22 417 210–1264	.226^b^
**Summary of comorbidities**				
None, *n* (%)	9 (10.1)	6 (13.3)	3 (6.8)	.3645
One, *n* (%)	17 (19.1)	7 (15.5)	10 (22.7)	
2 or More, *n* (%)	63 (70.8)	32 (71.1)	31 (70.5)	
Clinical Comorbidities				
History or current tobacco use, *n* (%)	15 (16.9)	7 (15.5)	8 (18.1)	.7762
Overweight, *n* (%)	39 (43.8)	21 (46.6)	18 (40.1)	.3847
Obesity, *n* (%)	25 (28.1)	11 (24.4)	14 (31.8)	.4758
Hypertension, *n* (%)	18 (20.2)	11 (24.4)	7 (15.9)	.2640
Diabetes, *n* (%)	15 (16.9)	8 (17.7)	7 (15.9)	.7393
Dyslipidaemia, *n* (%)	15 (16.9)	11 (24.4)	4 (9.1)	.0418
Hypertriglyceridemia, *n* (%)	43 (48.3)	22 (48.8)	21 (47.7)	.7486
Coronary artery disease, *n* (%)	0 (0.0)	0 (0.0)	0 (0.0)	–
Congestive heart failure, *n* (%)	1 (1.1)	0 (0.0)	1 (2.3)	.3201
Chronic respiratory disease (emphysema), *n* (%)	2 (2.3)	1 (2.3)	1 (2.3)	.9869
Asthma, *n* (%)	4 (4.5)	0 (0.0)	4 (9.1)	.0429
Chronic liver disease (chronic hepatitis, cirrhosis), *n* (%)	0 (0.0)	0 (0.0)	0 (0.0)	–
Chronic kidney disease, *n* (%)	0 (0.0)	0 (0.0)	0 (0.0)	–
Cancer, *n* (%)	0 (0.0)	0 (0.0)	0 (0.0)	–
Immune deficiency (acquired or innate), *n* (%)	0 (0.0)	0 (0.0)	0 (0.0)	–
Symptoms				
Dyspnoea, *n* (%)	33 (37.1)	18 (40)	15 (34.1)	.564
Cough, *n* (%)	67 (75.2)	34 (75.6)	33 (75.0)	.952
Chest pain, *n* (%)	35 (39.3)	19 (42.2)	16 (36.4)	.572
Rhinorrhoea, *n* (%)	39 (43.8)	19 (42.2)	20 (45.5)	.759
Headache, *n* (%)	46 (51.7)	22 (48.9)	24 (54.5)	.593
Sore throat, *n* (%)	41 (46.1)	20 (44.4)	21 (47.7)	.756
Malaise, *n* (%)	54 (60.7)	27 (60.0)	27 (61.4)	.895
Arthralgia, *n* (%)	44 (49.4)	18 (40.0)	26 (59.1)	.072
Myalgia, *n* (%)	48 (53.9)	23 (51.1)	25 (56.8)	.589
Brain fog, *n* (%)	43 (48.3)	25 (55.6)	18 (40.9)	.167
Ageusia, *n* (%)	50 (56.2)	28 (62.2)	22 (50.0)	.8041
Anosmia, *n* (%)	47 (52.8)	27 (60.0)	20 (45.5)	.7651
Diarrhoea, *n* (%)	19 (21.3)	11 (24.4)	8 (18.2)	.471
Abdominal pain, *n* (%)	22 (24.7)	8 (17.8)	14 (31.8)	.125
Jaundice, *n* (%)	4 (4.5)	3 (6.7)	1 (2.3)	.317
Vomiting and nausea, *n* (%)	5 (5.6)	2 (4.4)	3 (6.8)	.627
Conjunctivitis, *n* (%)	20 (22.5)	9 (20.0)	11 (25.0)	.572
Cyanosis, *n* (%)	0 (0.0)	0 (0.0)	0 (0.0)	–

^a^T‐Student; ^b^Mann‐Whitney

BMI: body mass index; IQR: interquartile range; PTCI: polymerised type I collagen; pSO_2_: oxygen saturation; SD: standard deviation.

On day 1 after the last PTIC or placebo administration, the IP‐10 levels decreased 75% in the PTIC group (*p <* .001) and 40% in the placebo group (*p =* .015) vs. baseline; this reduction was greater in the former group than in the latter (*p =* .0047; Figure 1A and F). The IL‐8 (44%, *p =* .045), M‐CSF (25%, *p =* .041) and IL‐1Ra (36%, *p =* .05) levels were also decreased in PTIC group vs. baseline (Figure 1B–F). TRAIL levels were decreased in the placebo group (14%, *p =* .002) vs. baseline (Figures 1E and S2).

**FIGURE 1:**
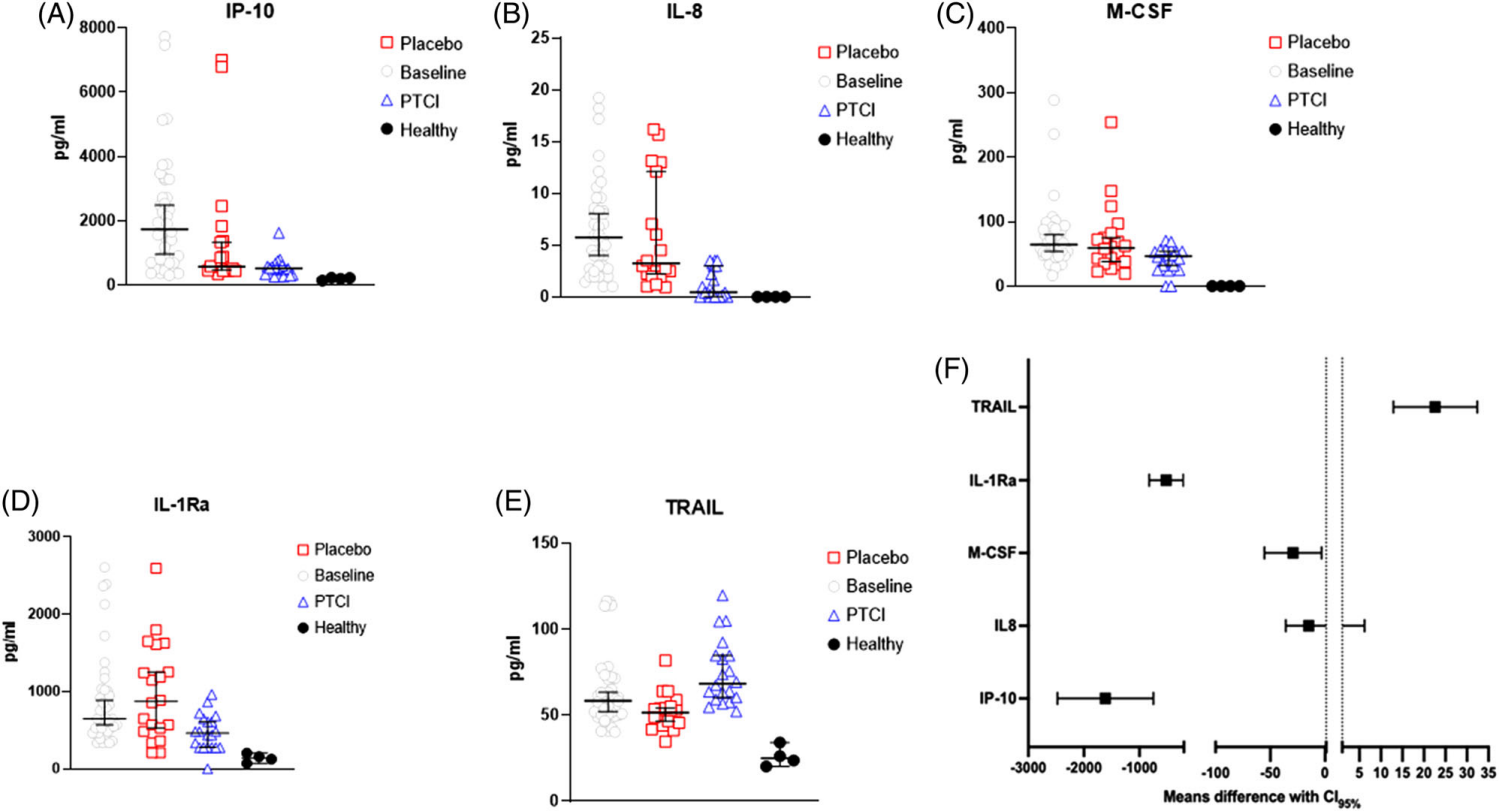
Serum cytokine and chemokine levels of SARS‐CoV2‐infected symptomatic outpatients at baseline and day 8 post‐treatment with PTIC or placebo. Data are expressed as median with 95% confidence. (A) IP‐10, IFN‐γ inducible protein‐10; (B) IL‐8, Interleukin‐8; (C) M‐CSF, Macrophage colony‐stimulating factor; (D) IL‐1Ra, IL‐1 receptor antagonist; (E) TRAIL, TNF‐related apoptosis inducing ligand; and (F) Forest plot (95% confidence intervals)

On days 1, 8 and 90 after the last PTIC or placebo administration, the patient percentage with oxygen saturation readings ≥92% in the PTIC and placebo groups were 90% vs. 67% (*p* = .007; mean oxygen saturation: 94 ± 2.4 vs. 93 ± 3.3, *p* = .085), 98% vs. 80% (*p* = .009; mean oxygen saturation; 95 ± 1.7 vs. 93 ± 2.2, *p* = .003) and 100% vs. 89% (*p* = .033; mean oxygen saturation: 95 ± 2.1 vs. 95 ± 2.3, *p* = .429), respectively (Table 2).

**TABLE 2:** Study endpoints

Characteristic	1 day post‐treatment with			8 days post‐treatment with			90 days post‐treatment with		
	PTIC (*N* = 44)	Placebo (*N* = 43)	*p* Value	PTIC (*N* = 42)	Placebo (*N* = 39)	*p* Value	PTIC (*N* = 40)	Placebo (*N* = 37)	*p* Value
SpO2 ≥ 92%, *n* (%)	40 (90.1)	29 (67.4)	.007	41 (97.6)	31 (79.5)	.009	40 (100)	33 (89.2)	.033
pSO_2_; mean ± SD Median IQR	94 ± 2.4 94 92–95	93 ± 3.3 93 91–95	.085	95 ± 1.7 95 93–96	93 ± 2.2 93 92–95	.003	95 ± 2.1 95 93–97	95 ± 2.3 95 93–97	.429
O2 supplementation, *n* (%)	2 (4.5)	4 (9.3)	.381	1 (2.3)	1 (2.6)	.958	0 (0.0)	0 (0.0)	‐
Inpatient admissions	0 (0.0)	3 (7.0)	.075	0 (0.0)	0 (0.0)	‐	0 (0.0)	0 (0.0)	‐
Symptoms									
Dyspnoea, *n* (%) Δ (%)	6 (13.6) –66.6	10 (25.6) –33.3	.166	3 (7.1) –83.3	9 (23.1) –40	.044	6 (15) –66.6	6 (16.2) –60	.883
Cough, *n* (%) Δ (%)	17 (38.6) –50	22 (56.4) –33.3	.105	11 (26.2) –67.6	21 (53.8) –36.3	.011	4 (10) –88.2	6 (16.2) –81.8	.418
Chest pain, *n* (%) Δ (%)	8 (18.2) –57.8	9 (23.1) –43.7	.581	5 (11.9) –73.6	6 (15.4) –62.5	.648	7 (17.5) –63.1	1 (2.7) –93.7	.033
Rhinorrhoea, *n* (%) Δ (%)	9 (20.5) –52.6	9 (41) –55.0	.772	6 (14.3) –68.4	6 (15.4) 0.0	.889	5 (12.5) –73.6	3 (8.1) –85.0	.528
Headache, *n* (%) Δ (%)	12 (27.3) –45.4	16 (41) –33.3	.186	9 (21.4) –59.0	15 (38.5) –37.5	.093	10 (25) –54.5	14 (37.8) –41.6	.224
Sore throat, *n* (%) Δ (%)	9 (30.5) –55.0	10 (25.6) –52.3	.575	5 (11.9) –75.0	6 (15.4) –71.4	.648	6 (15) –70.0	7 (18.9) –66.6	.646
Malaise, *n* (%) Δ (%)	16 (36.4) –40.7	18 (46.2) –33.3	.365	12 (28.6) –55.5	11 (28.2) –59.2	.971	11 (27.5) –59.2	8 (21.6) –70.3	.374
Arthralgia, *n* (%) Δ (%)	8 (18.2) –55.5	8 (20.5) –69.2	.788	6 (14.3) –66.6	6 (15.4) –76.9	.889	7 (17.5) –61.1	8 (21.6) –69.2	.648
Myalgia, *n* (%) Δ (%)	12 (27.3) –47.8	11 (28.2) –56.0	.925	5 (11.9) –78.2	6 (15.4) –76.0	.648	7 (17.5) –69.5	3 (8.1) –88.0	.221
Brain fog, *n* (%) Δ (%)	7 (15.9) –72.0	12 (30.8) –33.3	.108	6 (14.3) –76.0	7 (17.9) –61.1	.654	9 (22.5) –64.0	10 (27) –44.4	.645
Ageusia, *n* (%) Δ (%)	18 (40.9) –37.9	13 (33.3) –31.5	.476	11 (26.2) –62.0	8 (20.5) –57.8	.547	5 (12.5) –82.7	4 (10.8) –78.9	.818
Anosmia, *n* (%) Δ (%)	23 (52.3) 23.33	13 (33.3) 35.0	.082	16 (38.1) 46.6	9 (23.1) 55	.144	6 (15) 80.0	2 (5.4) 90.0	.168
Diarrhoea, *n* (%) Δ (%)	4 (9.1) –63.63	6 (15.4) –25	.379	3 (7.1) –72.7	2 (5.1) –75	.707	1 (2.5) –90.9	0 (0.0) –100.0	.333
Abdominal pain, *n* (%) Δ (%)	5 (11.4) –37.5	6 (15.4) –57.1	.590	0 (0.0) –100.0	3 (7.7) –78.5	*.067*	1 (2.5) –87.5	3 (8.1) –78.5	.268
Jaundice, *n* (%) Δ (%)	0 (0.0) –100.0	2 (5.1) 100.0	.128	0 (0.0) –100.0	0 (0.0) –100.0	‐	0 (0.0) –100	1 (2.7) 0.0	.295
Vomiting and nausea, *n* (%) Δ (%)	0 (0.0) –100.0	0 (0.0) –100.0		1 (2.4) –50	0 (0.0) –100.0	.332	0 (0.0) –100.0	0 (0.0) –100.0	‐
Conjunctivitis, *n* (%) Δ (%)	1 (2.3) –88.88	1 (2.6) –90.9	.931	1 (2.4) –88.8	1 (2.6) –90.9	.958	2 (5.0) –77.7	1 (2.7) –90.9	.603
Cyanosis, *n* (%) Δ (%)	0 (0.0) 0.0	1 (2.6) 100.0	.285	0 (0.0) 0.0	0 (0.0) 0.0	‐	0 (0.0) 0.0	0 (0.0) 0.0	‐

Δ: Delta calculated by taking: [(baseline data – day 1, 8 or 97 of follow‐up)/baseline data from table 1]×100. *p* value: PTCI vs. placebo.

IQR: interquartile range; PTCI: polymerised type I collagen; pSO_2_: oxygen saturation; SD: standard deviation.

The Kaplan–Meier survival curve for oxygen saturations ≥92% while breathing ambient air was statistically different between groups (log‐rank *p* = .0109; Figure 2A). Since there were no significant differences between groups at baseline, we did not make any adjustments. The Cox regression model indicated that the hazard for meeting an oxygen saturation lower than 92% was significantly lower in the PTIC than in the placebo group (HR 0.25, Wald *p* value = .0384). When stratifying by age, no changes occurred. Based on the accelerated time failure model, subjects of the PTIC group reached oxygen saturations 92% or greater 2.7‐fold faster than the placebo group at 3 and 8 days (*p* < .001 in both cases). In terms of risk, this implied that the PTIC group had a 63% lower risk for mean oxygen saturations readings below 92% (*p* < .001; Figure 2B).

**FIGURE 2:**
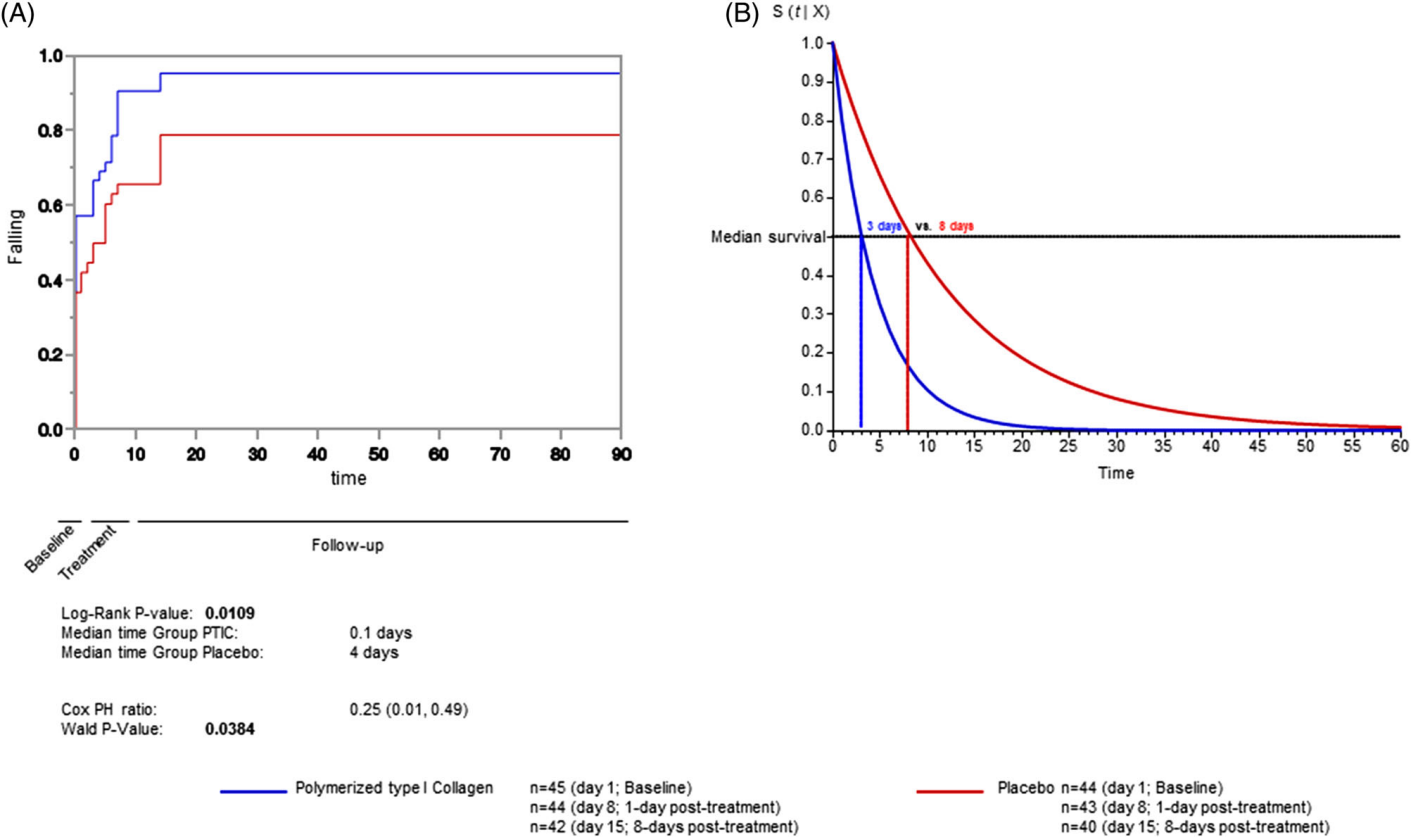
(A) Probability of oxygen saturation 92% or greater while breathing ambient air. (B) Accelerated time failure model for oxygen saturation 92% or greater while breathing ambient air among polymerised type I collagen and placebo

Symptom improvement was reported daily by every patient and compared with baseline. Symptom duration in the PTIC group was reduced by 6.1 ± 3.2 days vs. placebo (Figure S3 and Table 2).

At day 1 post‐treatment, 6/87 patients (7%) received supplemental oxygen via nasal cannula: 2/44 (4.5%) of the PTIC group (one patient received 2 L/min and another one received 3 L/min) and 4/43 (9.3%) of the placebo group (4–10 L/min). At day 8 post‐treatment, 2 of 81 patients (2.5%) received supplemental oxygen via nasal cannula: 1/42 (2.3%) of the PTIC group (one patient received 2 L/min) and 1/39 (2.6) of the placebo group (4 L/min). At day 90 post‐treatment, none of the patients required supplemental oxygen (Table 2).

At 1 day post‐treatment, 3/43 subjects (7%) of the placebo group were hospitalised for 5–21 days (Table 2). All patients were discharged alive, and no deaths occurred.

On days 1 and 8 post‐treatment with PTIC, serum levels of LDH and high sensitivity CRP (hs‐CRP) decreased (52% and 73%, respectively) vs. baseline levels (*p* = .002 and *p* < .001). In the placebo group, hsCRP levels were 3% and 67% lower at 1 and 8 days compared with baseline levels (Figure S4 and Table S3).

At days 1 and 8 post‐treatment, D‐dimer levels in PTIC subjects decreased (55% and 61%, respectively); in the placebo group, D‐dimer increased 42% and 32%, respectively Figure S4 and Table S3). No differences were detected in the other laboratory variables compared to the baseline.

No serious adverse events were detected (Table S1 and S2). PTIC was safe and well‐tolerated.

In summary, it has been demonstrated that intramuscular PTIC treatment of symptomatic COVID‐19 outpatients was useful for decreasing IP‐10, IL‐8 and M‐CSF, all of them biomarkers of severe disease,^10^ during the first week of treatment. It was associated with better oxygen saturation values when compared to placebo. Also, PTIC shortened symptom duration. On days 1 and 8 post‐treatment with PTIC, a higher mean oxygen saturation value and a higher proportion of patients retaining oxygen saturation values ≥92% were observed. This could be related to decreased dyspnoea, chest pain and cough. Regarding systemic inflammation, treatment with PTIC, statistically significant lower levels of hsCRP, D‐dimer and LDH, all of them identified as important biomarkers for the activity and severity of the disease, were observed. The benefit was evident in the early stage of the infection (7 days after symptom onset). PTIC was safe and well‐tolerated. It did not induce liver damage, impairment of haematopoiesis or alterations in blood count. We think that treating outpatients with PTIC could potentially avoid visits to the Emergency Department and hospitalisations. As judged by symptom improvement, it could aid in preventing sequelae, such as persistent dyspnoea.

## CONFLICT OF INTEREST

The authors declare that they have no competing interests.

## ROLE OF THE FOUNDING SOURCE

The funder of the study had no role in the study design, data collection, data analysis, data interpretation, or writing of the report. The corresponding authors had full access to all the data in the study and had final responsibility for the decision to submit for publication.

## Supporting information

SUPPORTING INFORMATION
