# Supplementary material for: Effect of polymerised type I collagen on hyperinflammation of adult outpatients with symptomatic COVID‐19
Source: Clin Transl Med. 2022 Mar 16;12(3):e763. doi: 10.1002/ctm2.763 (PMC8926898; doi:10.1002/ctm2.763)
Supplement: Supplementary file 1 — SUPPORTING INFORMATION [file CTM2-12-e763-s001.docx]

**Methods**

**Study design**

This was a single center, double-blind, placebo-controlled, randomized clinical trial that compared PTIC to placebo in adult outpatients with confirmed COVID-19. The study was approved by the institutional review board of the Instituto Nacional de Ciencias Médicas y Nutrición Salvador Zubirán (INCMNSZ, reference no. IRE 3412-20-21-1; supplement 1) and was conducted in compliance with the Declaration of Helsinki,^20^ the Good Clinical Practice guidelines, and local regulatory requirements. All participants provided written informed consent. This study is registered with the ClinicalTrials.gov identifier NCT04517162.

Trial candidates were identified in a prospective database of patients that went to a medical appointment at the hospital and were discharged home with a diagnosis of COVID-19 and symptomatic treatment. Diagnosis was based on suggestive symptoms (fever, headache, cough or dyspnea, plus at least another symptom such as malaise, myalgias, arthralgias, rhinorrhea, throat pain, conjunctivitis, vomiting or diarrhea) and a positive real-time reverse-transcription polymerase chain reaction result for SARS-CoV-2.

Staff reached candidates via telephone calls and informed them about the purpose of the study. Once in the hospital study site, inclusion and exclusion criteria were verified. Subjects that fulfilled the criteria mentioned above were included. Exclusion criteria were: hypersensitivity to PTIC or any of its excipients; COVID-19 patients that required hospitalization; all pregnant or breast-feeding women; patients with chronic kidney disease (estimated glomerular filtration rate less than 60 for more than 3 months or need for hemodialysis or hemofiltration); decompensated liver cirrhosis; congestive heart failure (New York Heart Association class III or IV); and patients with cerebrovascular disease, autoimmune disease, cancer, multiorgan failure or immunocompromise (solid organ transplant recipient or donor, bone marrow transplant recipient, AIDS, or treatment with biologic agents or corticosteroids). Study subjects were recruited between August 31 and November 7, 2020. Patients signed the informed consent before being randomly allocated to either PTIC or matching placebo.

During the first day of enrolment, candidates received a study kit that consisted of PTIC or placebo, a pulse oximeter, and a symptom questionnaire booklet. Patients were instructed on how to use the study medication and the pulse oximeter, and how to complete the questionnaires. Also, staff administered the first dose of PTIC or placebo on site, then participants self-administered their assigned treatment on their own.

Phone calls were made daily during the first 3 days of the trial to address participants’ questions, address any medication-related issues, and encourage completion of questionnaires daily. Additional phone calls were conducted on a case-by-case basis when participant’s survey data indicated values outside of the expected ranges. For participants that had a worsening disease course (89% or lower oxygen saturation while breathing ambient air), study staff recommended that medical attention be provided in the Emergency Department at no cost. If the patient required hospitalization and treatment with dexamethasone, then he/she was eliminated from the study. However, these patients were included in the intention-to-treat analysis. Patients were evaluated by staff at the study site (S.M-F, A.P-R, D.A-Ll, H.O-P, E.O-H, E.R-C.) on days 8, 15 and 97 (1, 7 and 90 days after the last dose of PTIC or placebo, respectively), and patients were encouraged to complete questionnaires daily.

**Participants and data collection**

The study included non-hospitalized adults with COVID-19 whose symptoms started within the previous 7 days before randomization (Figure 1). Individuals were asked to provide personal information (date of birth, type of job, educational level, previous contact with infected individuals), pre-existing conditions (systemic hypertension, diabetes mellitus, cardiovascular disease, cerebrovascular disease, hypertriglyceridemia, dyslipidemia) and symptoms. Personal data, exposure history, clinical presentation, chest computed (CT) tomography, laboratory tests, previous treatment and outcome data were collected both prospectively and from inpatient medical records. Laboratory data collected from each patient from study days 1 (baseline), 8 (day 1 posttreatment), 15 (day 8 posttreatment) and 97 (day 90 posttreatment) included complete blood count, coagulation profile, serum biochemical tests (including renal and liver function tests, electrolytes, lactate dehydrogenase, D dimer and creatine kinase), serum ferritin, C-reactive protein (CRP) and procalcitonin. Chest CT scans were done in all patients at baseline.

**Study sample**

According to a study published by Yang Y. et al,^21^ IFN-γ-inducible protein 10 (IP-10), a host protein involved in lung injury from virus-induced hyperinflammation, is highly associated with disease severity. IP-10 predicts the progression of COVID-19 and its reduction by 50% avoids the development of critical or severe disease. Thus, it was arbitrarily decided that a difference of 50% on this biomarker on day 8 (day 1 posttreatment) vs baseline would be significant. Assuming this difference as the effect size, two-tailed alpha=0.05, power=0.80 and Cohen's d = 0.61522 and losses to follow-up, the sample size was set at 45 patients in each group. To calculate the sample size, the R software (version 3.6.2) with the “pwr” package was used.

**Randomization**

Patients were randomized in a 1:1 fashion to PTIC or placebo. The Excel program displayed a random allocation list to the laboratory manager (K.R-R) who was in charge of assigning PTIC or placebo to study subjects. All outcome assessors, investigators and research staff who were in contact with participants were blinded to participant treatment assignment.

**Intervention**

Participants received an intramuscular dose of either PTIC (1.5 ml, equivalent to 12.5 mg of collagen) every 12 h for 3 days and then every 24 h for 4 days, or placebo. Only acetaminophen or acetylsalicylic acid were allowed as concomitant therapy. Monitoring of compliance was evaluated by counting empty vials returned on subsequent visits.

**Measurement of serum cytokines**

The blood samples were taken from study subjects at baseline and day 8 (1 day after the last dose of PTIC or placebo, respectively) and placed in serum separator tubes, followed by five tube inversions. The samples were allowed to stand for 20 minutes and were then centrifuged at 2000 rpm at 4°C. The sera were aspirated and separated into 300 μl aliquots. After collection, the serum samples were stored at -70°C. The concentrations of cytokines and chemokines were determined with the Hu Cyto screening panel 48-Plex kit (Bio-Rad, Berkeley, Calif) as reported elsewhere.^22^ According to their functions, the cytokines/chemokines were grouped into 6 categories: interleukins (IL-1α, IL-1β, IL-1Ra, IL-2, IL-2Rα, IL-3, IL-4, IL-5, IL-6, IL-7, IL-8, IL-9, IL-10, IL-12p40, IL-12p70, IL-13, IL-15, IL-16, IL-17, and IL-18), colony stimulating factors (G-CSF, GM-CSF, M-CSF, and stem cell factor [SCF]), interferons (IFN-α_2_ and IFN-γ), tumor necrosis factor (TNF-α, TNF-β, and TNF-related apoptosis inducing ligand [TRAIL]), growth factors (basic fibroblast growth factor [FGF], nerve growth factor-β [NGF-β], hepatocyte growth factor [HGF], leukemia inhibitory factor [LIF], platelet-derived growth factor [PDGF-BB], vascular endothelial growth factor [VEGF], and stem cell growth factor-β [SCGF-β]), and chemokine family (cutaneous T-cell-attracting chemokine [CTACK], eotaxin, growth related oncogene-α [GRO-α], IFN-γ inducible protein-10 [IP-10], monocyte chemoattractant protein-1 [MCP-1], MCP-3, migration inhibitory factor [MIF], monokine induced by interferon-γ [MIG], macrophage-inflammatory protein [MIP-1α], MIP-1β, regulated upon activation normal T-cell expressed and secreted [RANTES], and stromal cell-derived factor-1α [SDF-1α]).

**Primary and secondary outcomes**

The primary outcome was a mean reduction of at least 50% in the level of IP-10 compared to baseline. The secondary outcomes were an oxygen saturation of 92% or greater while breathing ambient air and duration of symptoms. Grading of symptoms was as follows: 0= absent, 1= mild, 2= moderate and 3= severe (continuous variable). Two comparisons were made regarding symptom duration and intensity: 1) at 8, 15 and 97 days as compared to baseline in each group, and 2) PTIC vs placebo.

The secondary outcomes were measured using participants’ self-reported responses during the next 8, 15 and 97 days after randomization. Each participant’s diary and health status were further checked by phone contact and by physician in-person evaluation on days 8, 15 and 97.

Adverse events and serious adverse events were intentionally sought by participants and study staff during the first 97 days after first dose of either PTIC or placebo (supplement 2).

**Chest CT**

A semi-quantitative scoring system was used to estimate the pulmonary involvement based on the pulmonary affected area.^23,24^

**Severity of the Guangzhou score**

The occurrence of critical illness was determined according to the Guangzhou score.^25^

**Statistical analysis**

A descriptive analysis was done: continuous variables were expressed by means and standard deviations (normal distribution) or medians and interquartile ranges (non-normal distribution), and categorical variables were summarized using proportions. The comparison of continuous variables between two groups was performed using the Wilcoxon rank sum test. For evaluation of the primary and secondary outcomes, intention-to-treat inferential analyses were done. Variables with a significant deviation from normality (set at the 0.05 level) in the Kolmogorov – Smirnov test were transformed to a logarithmic scale. In this comparison, the average differences represented the percentage of change (delta) between posttreatment status vs pretreatment status in each study group. For continuous variables with normal distribution, delta was calculated by subtracting the pretreatment value from the posttreatment value (on days 8, 15 or 97) and dividing by the pretreatment value. Either Student’s t test, Wilcoxon rank sum test or Chi-squared (Pearson’s) test were used for the intention-to-treat inferential analysis of normal or transformed (normalized) continuous variables, non-normally distributed continuous variables, and categorical variables, respectively. Based on the post-intervention evaluation intervals (on days 8, 15 or 97), we implemented an exponential model of Accelerated Time Failure in SAS v 9.4. We adopted this approach based on the shape and scale parameters of the latter (δ ≈ σ ≈ 1, respectively). In addition, we analyzed the other risk distributions and further corroborated the goodness of fit of the exponential by means of likelihood ratio tests, the Akaike information criterion (AIC), and the examination of logarithmic survival plots. For the Kaplan-Meier curves, we modelled oxygen saturation ≤92%-free survival in the PRISM GraphPad Statistics analysis program. To bolster our approach, we estimated the survivor function for the probability of achieving >92% oxygen saturation while breathing ambient air by the Kaplan-Meier method. Significant differences in survivor experience across the follow-up were corroborated with a log-rank test. Finally, we estimated the hazard for not meeting the primary outcome by Cox regression. Our data and model hold the proportionality of the hazard’s assumption.

| T A B L E S1. Non-serious adverse events | | |
| --- | --- | --- |
| **MedDRA System Organ Class** | **Preferred Term** |  |
| Any System Organ Class | Any Preferred Term |  |
| Blood and lymphatic system disorders | Anemia |  |
|  | Lymphopenia |  |
| Cardiac disorders | Atrial fibrillation |  |
| General disorders and administration site conditions | Pyrexia |  |
| Infections and infestations | Pneumonia |  |
| Investigations | Hemoglobin decreased |  |
|  | Glomerular filtration rate decreased |  |
|  | Aspartate aminotransferase increased |  |
|  | Lymphocyte count decreased |  |
|  | Blood glucose increased |  |
|  | Alanine aminotransferase increased |  |
|  | Blood bilirubin increased |  |
|  | Blood creatinine increased |  |
|  | Prothrombin time prolonged |  |
|  | Blood albumin decreased |  |
|  | Transaminases increased |  |
|  | Creatinine renal clearance decreased |  |
| Metabolism and nutrition disorders | Hyperglycemia |  |
|  | Acidosis |  |
|  | Hypoalbuminemia |  |
|  | Alkalosis |  |
| Psychiatric disorders | Delirium |  |
| Renal and urinary disorders | Acute kidney injury |  |
| Respiratory, thoracic and mediastinal disorders | Hypoxia |  |
|  | Dyspnea |  |
|  | Respiratory distress |  |
| Uncoded | Uncoded |  |
| Vascular disorders | Hypotension |  |
|  | Hypertension |  |
|  | Deep vein thrombosis |  |

| T A B L E S2. Serious adverse events | | |
| --- | --- | --- |
| **MedDRA System Organ Class** | **Preferred Term** |  |
| Any System Organ Class | Any Preferred Term |  |
| Cardiac disorders | Cardiac arrest |  |
|  | Atrial fibrillation |  |
| Infections and infestations | Septic shock |  |
|  | Pneumonia viral or bacterial |  |
| Investigations | Glomerular filtration rate decreased |  |
| Renal and urinary disorders | Acute kidney injury |  |
| Respiratory, thoracic and mediastinal disorders | Respiratory failure |  |
|  | Acute respiratory failure |  |
|  | Respiratory distress |  |
|  | Hypoxia |  |
|  | Pneumothorax |  |
|  | Pulmonary embolism |  |
| Surgical and medical procedures | Mechanical ventilation |  |
|  | Endotracheal intubation |  |
| Uncoded | Uncoded |  |
| Vascular disorders | Hypotension |  |
|  | Shock |  |

| **T A B L E S3 Follow-up of laboratory tests** | | | | | | | | | |
| --- | --- | --- | --- | --- | --- | --- | --- | --- | --- |
| **Characteristic** | **1-day posttreatment with** | | | **8-days posttreatment with** | | | **90-days posttreatment with** | | |
|  | **PTIC**  **(N= 44)** | **Placebo**  **(N= 43)** | ***P*- value** | **PTIC**  **(N=42)** | **Placebo**  **(N= 40)** | ***P*-value** | **PTIC**  **(N= 40)** | **Placebo**  **(N= 37)** | ***P*-value** |
| **Complete blood count** |  |  |  |  |  |  |  |  |  |
| Leukocyte count (x10^3/µL), mean ± SD  Median  IQR  Δ (%) | 6.65 ± 1.92  6.45  4.90-7.70  14.08 | 6.01 ± 1.51  5.60  4.90-6.90  5.34 | 0.108^b^ | 6.41 ± 1.42  6.35  5.35-7.32  10 | 6.47 ±1.60  6.25  5.40-7.50  13.50 | 0.926^b^ | 6.71 ± 1.31  6.65  5.8-7.8  -15.15 | 6.32 ± 1.43  6.15  5.20-7.02  -10.79 | 0.079 ^b^ |
| Hemoglobin (g/dL), mean ± SD  Median  IQR  Δ (%) | 15.04 ± 1.59  14.80  14.02-16  -2.94 | 15.27 ± 1.77  15  14.40-16.70  -1.15 | 0.472^a^ | 14.83 ± 1.53  15.15  13.80-16.02  -4.26 | 15.02 ± 1.50  14.85  14.20-16.35  -2.78 | 0.534^b^ | 15.16 ± 1.75  15.40  14-16.40  2.14 | 16.20 ± 5.01  15.35  14.30-16.75  -4.84 | 0.418 ^b^ |
| Platelets (K/µL), mean ± SD  Median  IQR  Δ (%) | 305.82 ± 101.21  276.50  235-343.25  8 | 329.58 ± 112.87  310  256-402  24.74 | 0.190^b^ | 287.95 ± 81.10  269.50  222.75-341.25  1.69 | 339.18 ± 122.03  314.50  274-404  28.38 | 0.030^b^ | 275.26 ± 80.22  263.50  219.75-305.50  2.8 | 284.60 ± 83  257.50  233.50-224.25  -7.72 | 0.640 ^b^ |
| Lymphocyte count (%), mean ± SD  Median  IQR  Δ (%) | 29.73 ± 8.45  30.95  24.35-34.72  -1.38 | 30.36 ± 9.46  30.80  24.80-36.50  -0.77 | 0.595^a^ | 31.21 ± 8.18  31.30  26.17-36.50  3.52 | 32.25 ± 7.36  32.25  27.20-39.15  7.05 | 0.677^a^ | 31.54 ± 6.92  32.45  26.25-36.32  -6.08 | 32.92 ± 6.58  31.45  29.10-37.77  -8.44 | 0.837 ^a^ |
| Neutrophil count (%), mean ± SD  Median  IQR  Δ (%) | 60.76 ± 8.87  57.70  54.32-66.40  1.45 | 59.62 ± 10.17  59.30  52.10-66.20  -1 | 0.310^a^ | 58.90 ± 8.39  57.10  54-64.27  -1.66 | 57.66 ± 7.60  57.75  50.52-65.07  -4.25 | 0.830^a^ | 58.80 ± 6.69  57.90  54-64.52  -4.62 | 57.25 ± 6.77  57.65  53.35-61.75  -9.27 | 0.892 ^a^ |
| Neutrophil-lymphocyte ratio (NLR), mean±SD  Median  IQR  Δ (%) | 2.50 ± 1.98  1.89  1.58-2.70  -4.71 | 2.58 ±2.45  1.91  1.42-2.67  1.90 | 0.674^b^ | 2.14 ± 1.10  1.85  1.48-2.37  -18.48 | 1.97 ± 0.90  1.80  1.28-2.40  -22.06 | 0.464^b^ | 2.01 ± 0.75  1.79  1.53-2.47  1.82 | 1.85 ± 0.63  1.78  1.40-2.15  4.93 | 0.526 ^b^ |
| **Liver function test (LFT)** |  |  |  |  |  |  |  |  |  |
| Total bilirubin (mg/dL), mean ± SD  Median  IQR  Δ (%) | 0.72 ± 0.28  0.67  0.49-0.85  15.66 | 0.65 ± 0.28  0.64  0.42-0.71  4.46 | 0.276^b^ | 0.72 ± 0.32  0.63  0.47-0.91  15.93 | 0.71 ± 0.40  0.65  0.50-0.75  15.22 | 0.893^b^ | 0.74 ± 0.34  0.70  0.48-0.84  23.40 | 0.70 ± 0.26  0.68  0.52-0.89  26.88 | 0.284 ^a^ |
| Direct bilirubin (mg/dL), mean ± SD  Median  IQR  Δ (%) | 0.13 ± 0.07  0.11  0.09-0.14  2.44 | 0.13 ± 0.06  0.12  0.08-0.15  9.84 | 0.712^b^ | 0.13 ± 0.06  0.11  0.09-0.14  -0.95 | 0.12 ± 0.05  0.11  0.09-0.14  -12.27 | 0.937^b^ | 0.13 ± 0.06  0.12  0.09-0.14  -19.32 | 0.12 ± 0.08  0.12  0.10-0.15  -13.07 | 0.754 ^b^ |
| Indirect bilirubin (mg/dL), mean ± SD  Median  IQR  Δ (%) | 0.59 ± 0.22  0.55  0.40-0.69  18.81 | 0.52 ± 0.24  0.49  0.34-0.58  7.02 | 0.129^b^ | 0.59 ± 0.28  0.52  0.38-0.73  20.23 | 0.59 ± 0.35  0.53  0.42-0.64  21.42 | 0.952^b^ | 0.61 ± 0.29  0.55  0.38-0.70  -2.44 | 0.57 ± 0.22  0.54  0.43-0.73  14.41 | 0.297 ^a^ |
| Aminotransferase, serum aspartate (AST) (U/L), mean ± SD  Median  IQR  Δ (%) | 24.94 ± 12.21  21.50  17-27  12.15 | 27.79 ± 20.06  21.50  17.75-29.25  -17.93 | 0.542^b^ | 22.02 ± 13.48  19  15-23  -22.43 | 23.13 ± 14.18  20  15-23.75  **-31.72** | 0.552^b^ | 21.29 ± 9.43  20  16-24  -23.66 | 23.94 ± 13  21  18.75-24.50  -17.26 | 0.230 ^b^ |
| Aminotransferase, serum alanine (ALT) (U/L), mean ± SD  Median  IQR  Δ (%) | 33.50 ± 22.39  30  14-45.50  6.9 | 35.32 ± 28.67  28.50  19.75-47.50  9.99 | 0.631^b^ | 25.55 ± 14.90  22.50  14-32.70  28.31 | 31.05 ± 29.14  26.50  18-37.50  20.81 | 0.330^b^ | 24.59 ± 15.25  22  13.50-25.50  31 | 27.39 ± 12.70  24  19.50-31.75  30.20 | 0.089 ^b^ |
| Albumin (g/dL), mean ± SD  Median  IQR  Δ (%) | 4.27 ± 0.57  4.31  3.96-4.65  -2.75 | 4.28 ± 0.43  4.33  3.90-4.61  -0.92 | 0.549^a^ | 4.41 ± 0.45  4.37  4.36-4.71  -0.22 | 4.34 ± 0.35  4.33  4.06-4.58  -0.59 | 0.360^a^ | 4.53 ± 0.35  4.54  4.31-4.80  25.01 | 4.47 ± 0.30  4.49  4.25-4.66  29.31 | 0.455 ^a^ |
| **Fasting glucose (mg/dL)**  Mean ± SD  Median  IQR  Δ (%) | 117.80 ± 68.23  96  88.25-113.25  1.78 | 105.64 ± 39.59  95  86.75-103.25  9.51 | 0.397^b^ | 115.33 ± 76.88  93  88-116.5  -3.33 | 103.88 ± 41.85  93  88.25-99.75  -8.99 | 0.556^b^ | 108.26 ± 55.34  95.50  87-104  -3.06 | 106.89 ± 40.08  96  89.75-106  -3.57 | 0.629 ^b^ |
| **Lactate dehydrogenase (LDH) (U/L)**  Mean ± SD  Median  IQR  Δ (%) | 145.11 ± 42.23  138.50  122.50-156.25  -12.10 | 165.74 ± 50.58  155  134-180  -1.54 | 0.007^b^ | 135.02 ± 22.86  134  119.65-152  -18.21 | 156.55 ± 53.32  147  128-172  -7 | 0.025^b^ | 149.73 ± 25.08  146.50  130.50-164  9.26 | 159.21 ± 20.58  158  144.75-175.25  6.35 | 0.302 ^a^ |
| **C-reactive protein (high sensitivity) (mg/dL)**  Mean ± SD  Median  IQR  Δ (%) | 0.63 ± 0.99  0.31  0.11-0.67  **-52.27** | 1.90 ± 3.99  0.32  0.13-1.83  -2.46 | 0.359^b^ | 0.35 ± 0.59  0.17  0.10-0.31  **-73.32** | 0.63 ± 1.48  0.23  0.11-0.46  **-66.83** | 0.294^b^ | 0.42 ± 1.07  0.13  0.09-0.40  68.07 | 0.36 ± 0.42  0.18  0.09-0.46  82.54 | 0.423 ^b^ |
| **Ferritin (ng/mL)**  Mean ± SD  Median  IQR  Δ (%) | 201.97 ± 239.76  119.15  44.95-271.60  -14.11 | 240.31 ± 275.79  173  61.10-315.80  -4.63 | 0.329^b^ | 146.99 ± 196.05  100.10  30.90-199.02  **-37.49** | 172.08 ± 217.63  136.95  44.92-210.87  **-31.71** | 0.466^b^ | 92.61 ± 95.88  69.60  23.05-130.62  60.62 | 77.70 ± 88.58  57  20.95-89.70  69.16 | 0.614 ^b^ |
| **D-dimer (ng/dL)**  Mean ± SD  Median  IQR  Δ (%) | 765.09 ± 1228.78  384  292-704  **-54.68** | 660.81 ± 1118.94  449  142-7634  41.52 | 0.879^b^ | 667.50 ± 994.12  373  231.75-728.50  **-61.47** | 614.25 ± 1183.50  409  243-417  31.55 | 0.973^b^ | 348.86 ± 294.71  266  188.25-443.25  79.86 | 335.31 ± 186.57  282  184.75-447.50  28.19 | 0.751 ^b^ |

Δ: Delta calculated by taking: [(Baseline data - Day 1, 8, or 97 of follow-up) / Baseline data from table 1]*100. ^a^T-Student; ^b^Mann-Whitney IQR: interquartile range; PTCI: polymerized type I collagen; pSO_2_: oxygen saturation; SD: standard deviation

**Figure S1.** Flow chart.


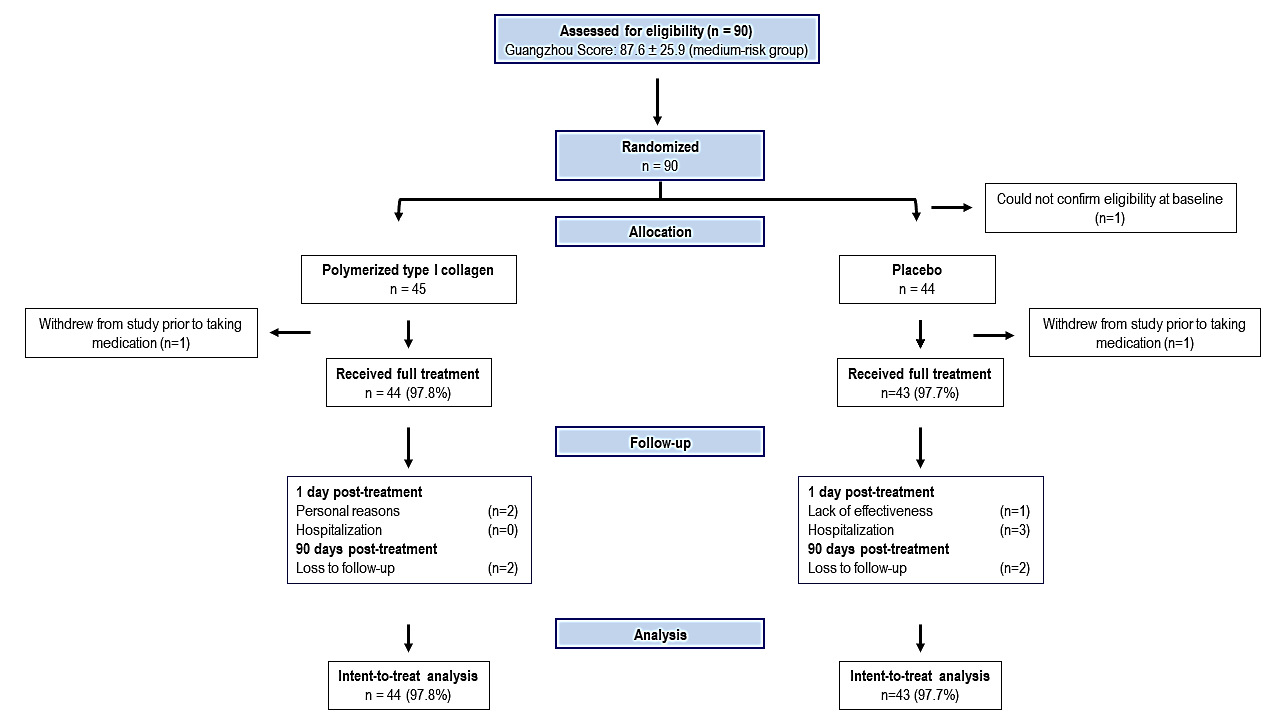


**Figure S2.** Serum cytokine and chemokine levels of SARS-CoV2-infected symptomatic outpatients at baseline and day 1 post-treatment with PTIC or placebo. Data are expressed as median with 95% confidence

**
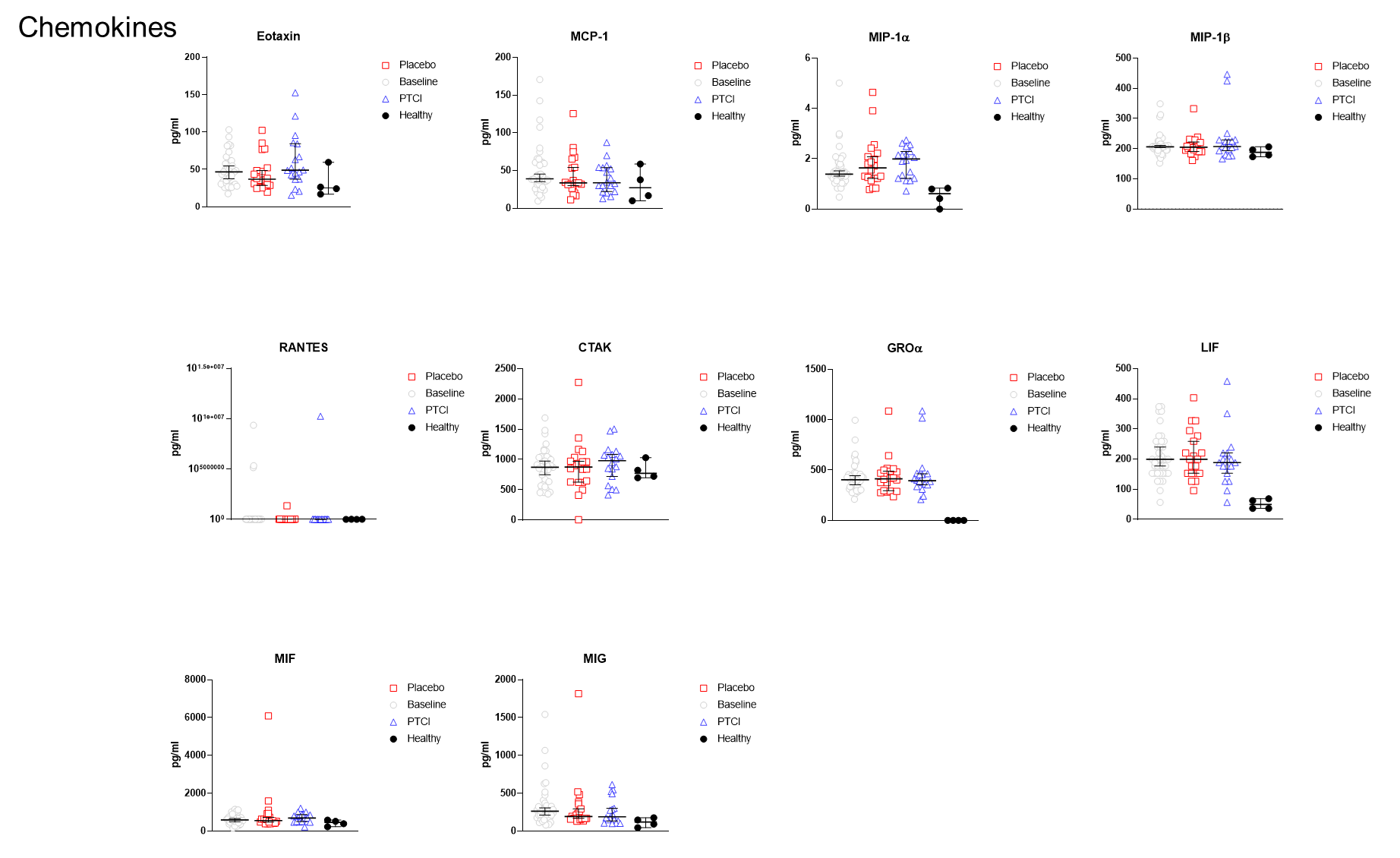
**


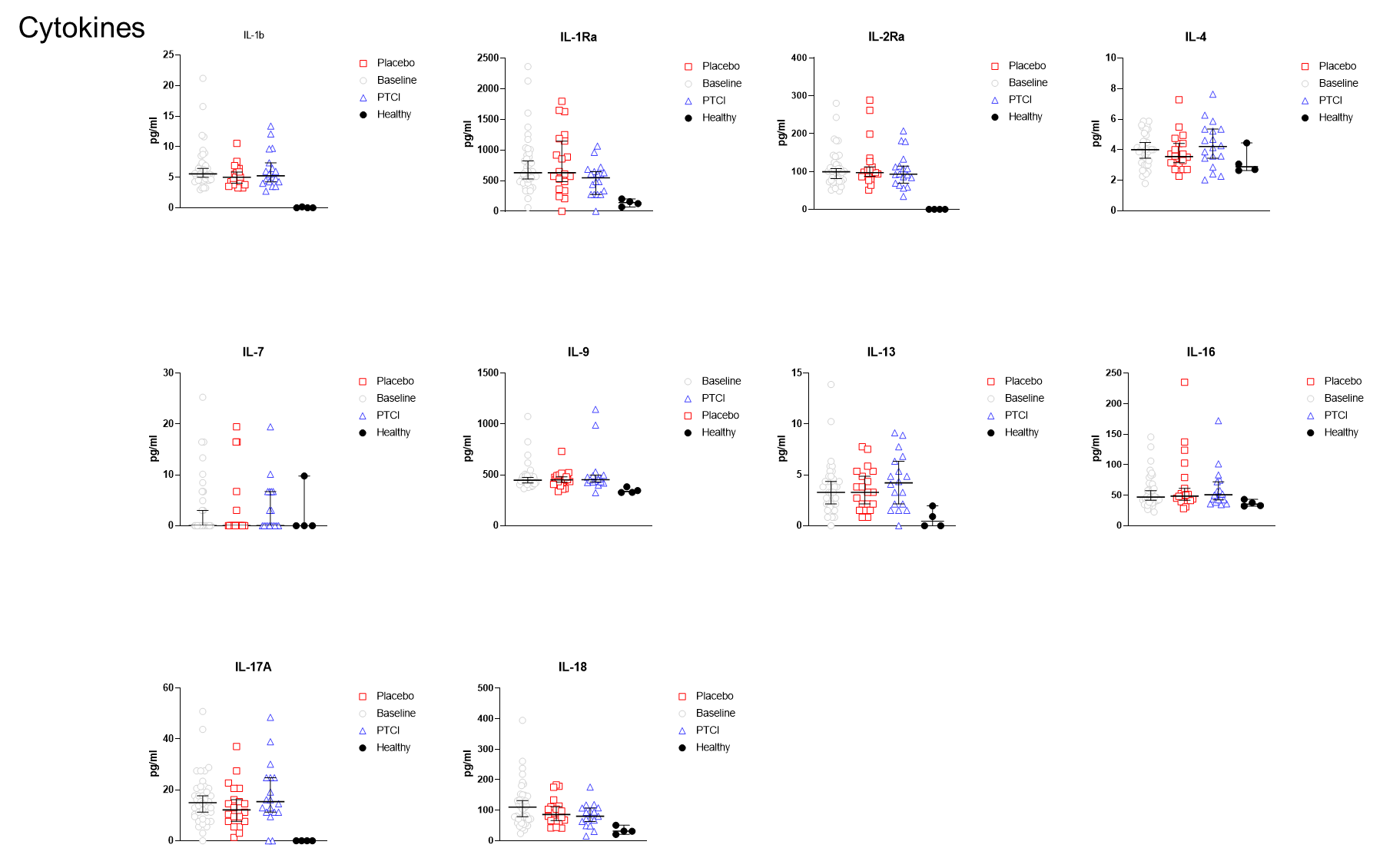


**
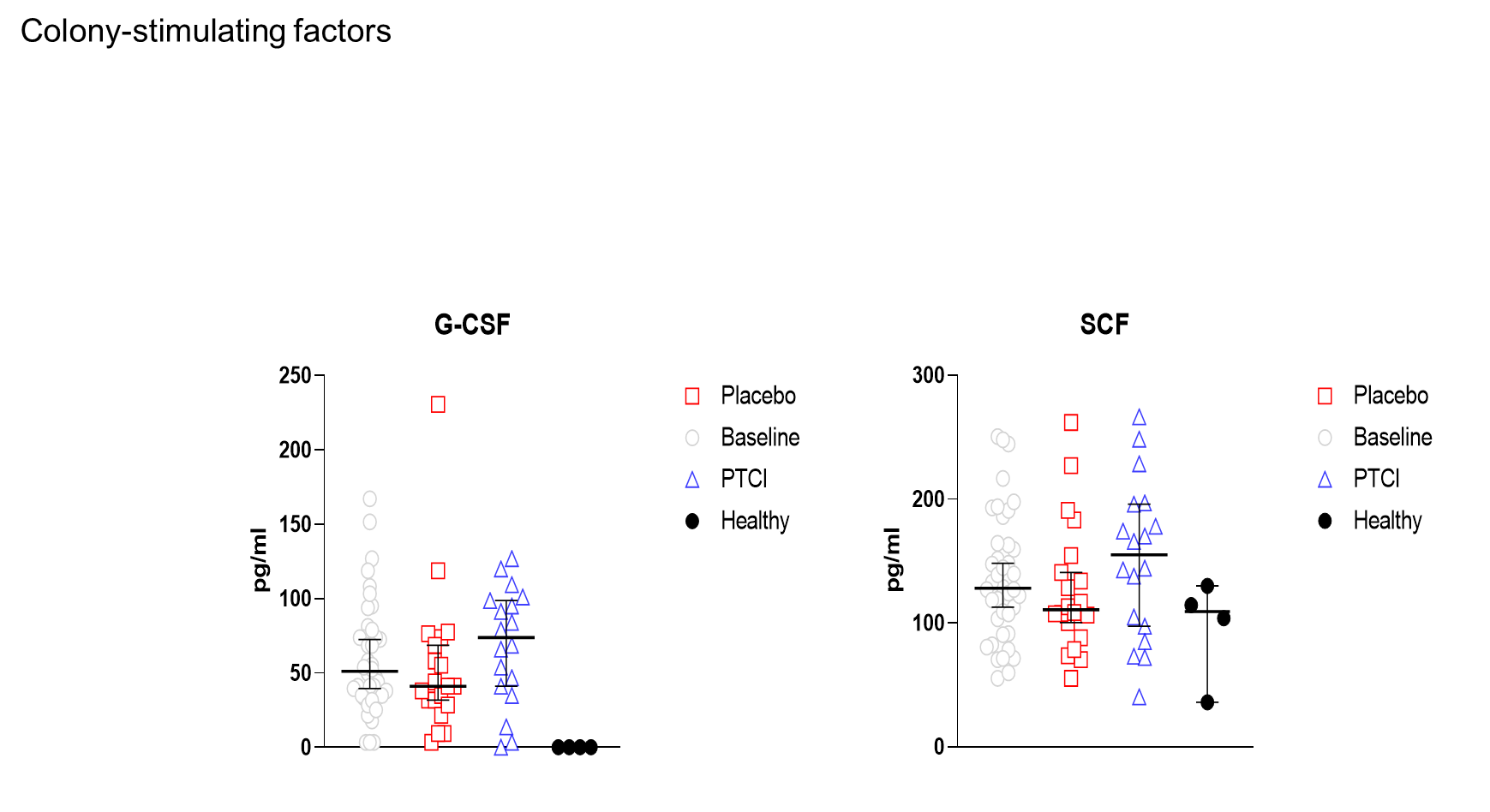
**

**
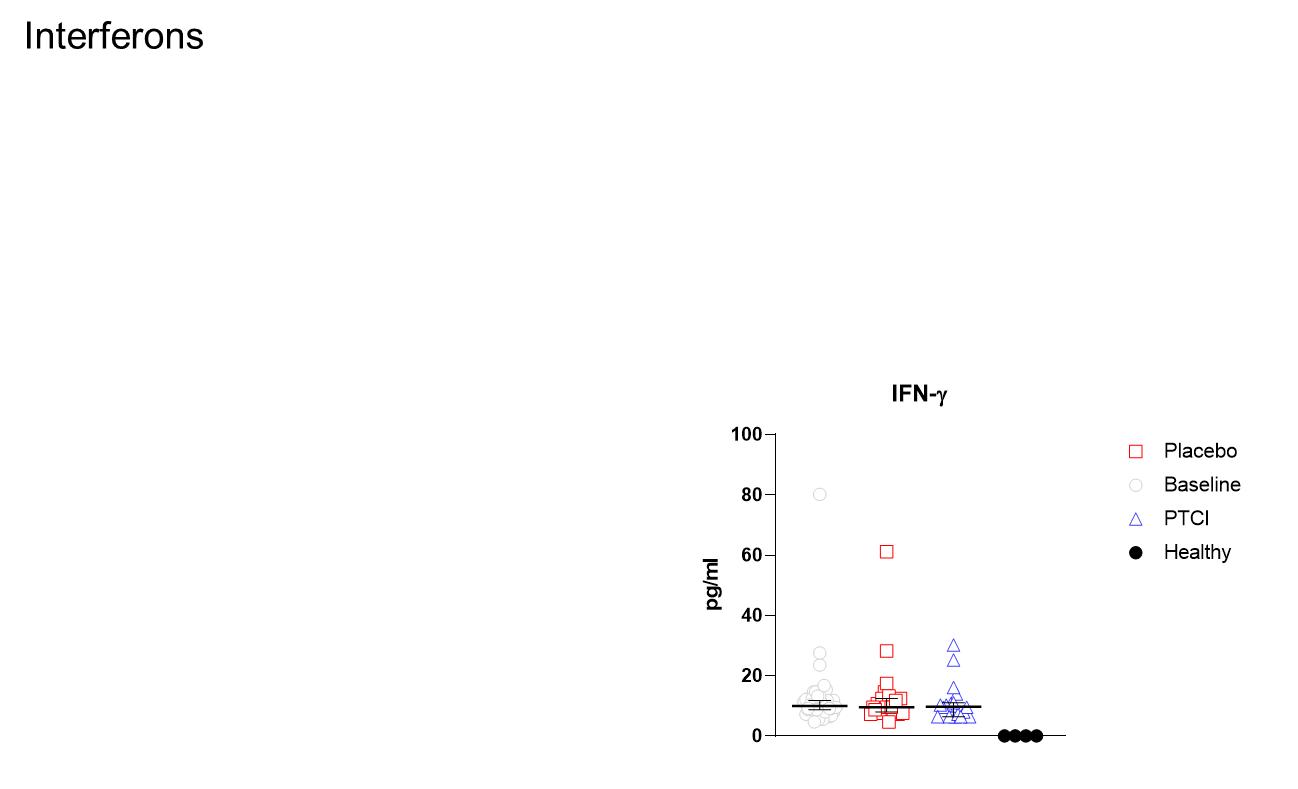
**

**
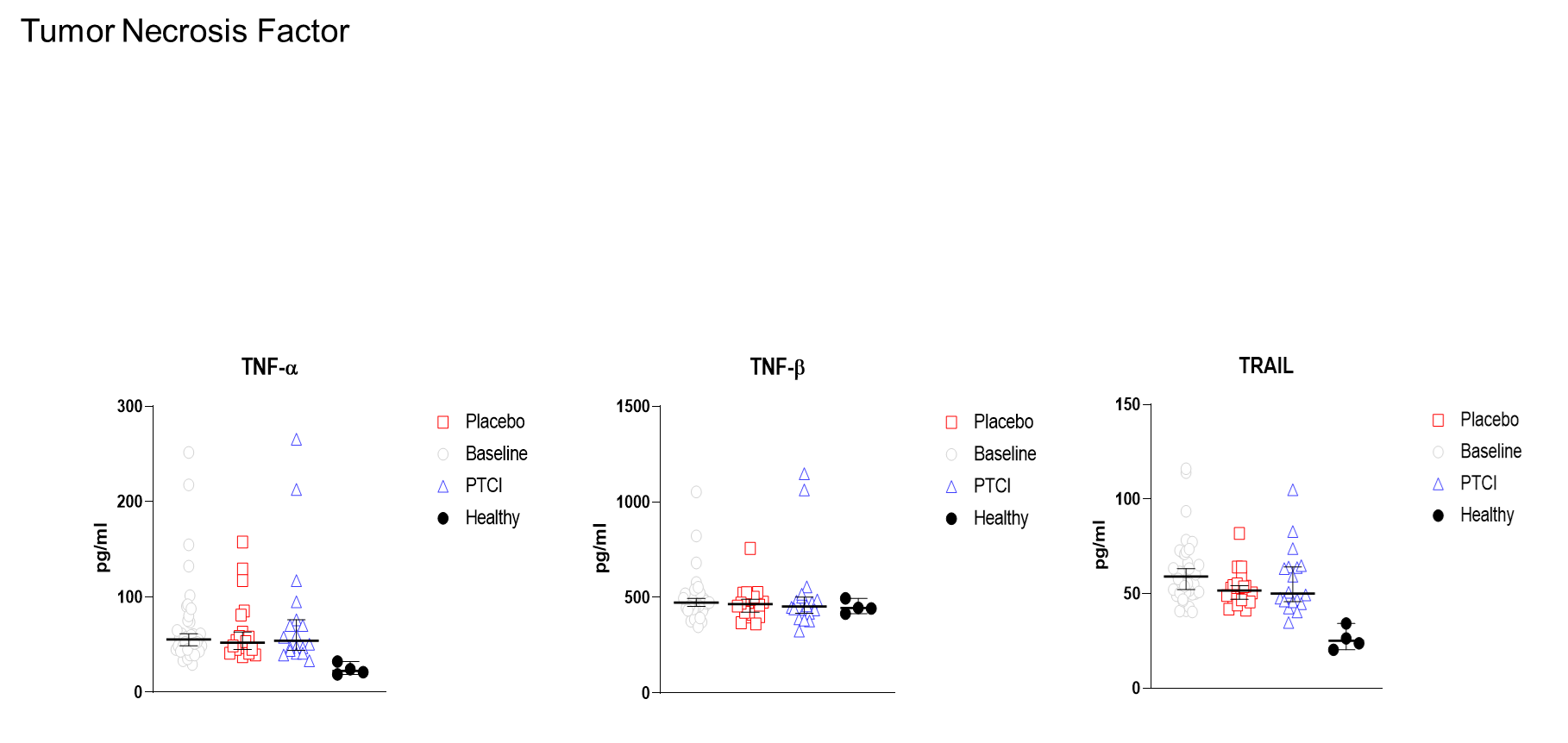
**

**
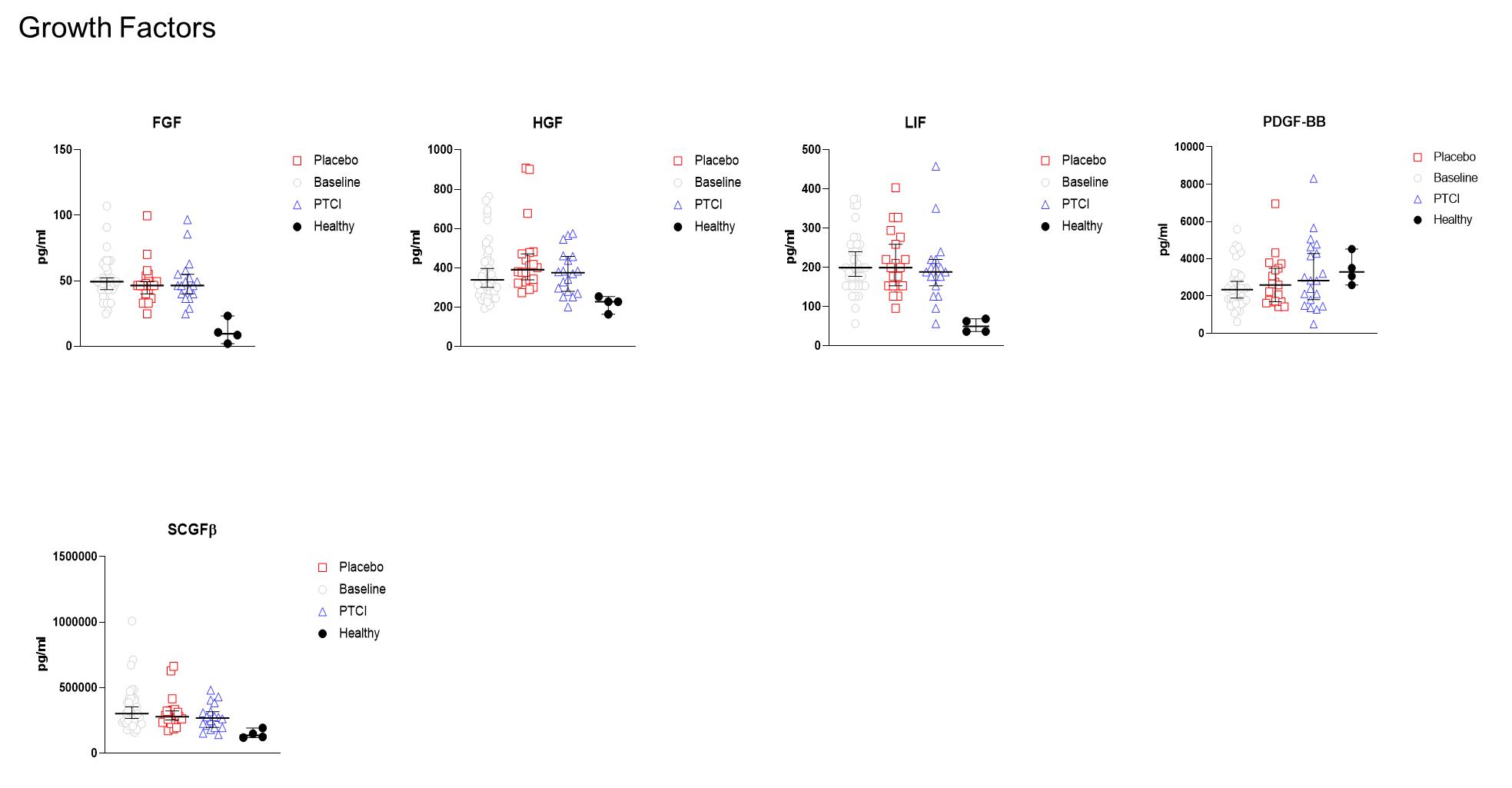
**

**Figure S3** Intensity of symptoms during treatment and follow-up of outpatients with symptomatic COVID-19 treated with polymerized type I collagen or placebo. (A) Dyspnea, (B) cough, (C) chest pain, (D) sore throat, (E) malaise, (F) arthralgia, (G) myalgia, (H) abdominal pain, (I) brain fog, (J) headache, (K) anosmia, and (L) ageusia. The intensity of the symptom was evaluated on a 4-point rating scale (0 = without symptom, 1 = mild, 2 = moderate, 3 = severe). Blue lines represent the group of patients under polymerized type I collagen treatment. Red lines represent the group of patients under placebo treatment. Results depict mean ± standard error of the mean. Blue arrows show the day in which the treatment reached a *P* < 0.05 compared to baseline for polymerized type I collagen treatment. Red arrows show the day in which the treatment reached a *P* < 0.05 compared to baseline for placebo.

**
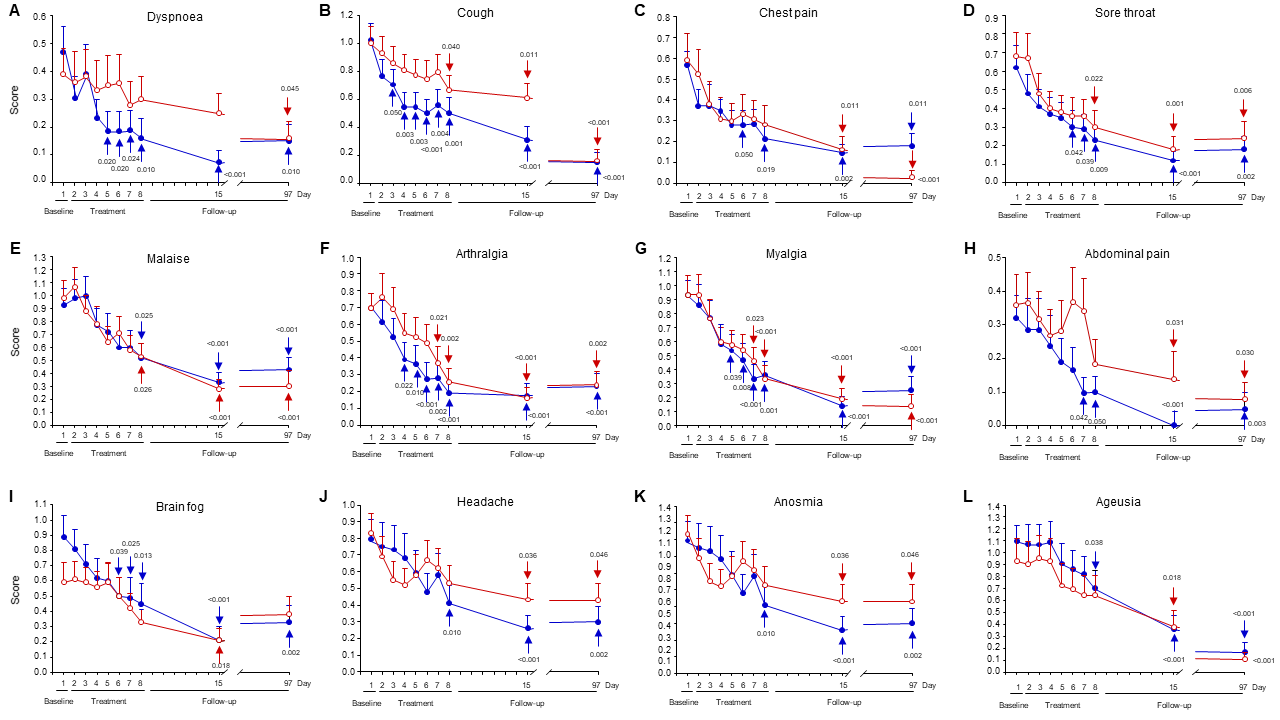
**

**Figure S4.** PCR and D-dimer during treatment and follow-up of outpatients with symptomatic COVID-19 treated with polymerized type I collagen or placebo. (A) high sensitivity C-reactive protein, and (B) D-dimer. Blue lines represent the group of patients under polymerized type I collagen treatment. Red lines represent the group of patients under placebo treatment. Results depict mean ± error standard of the mean. Blue and red arrows show *P-value* (Day 1, 8 and 97 days post-treatment compared to baseline).

**
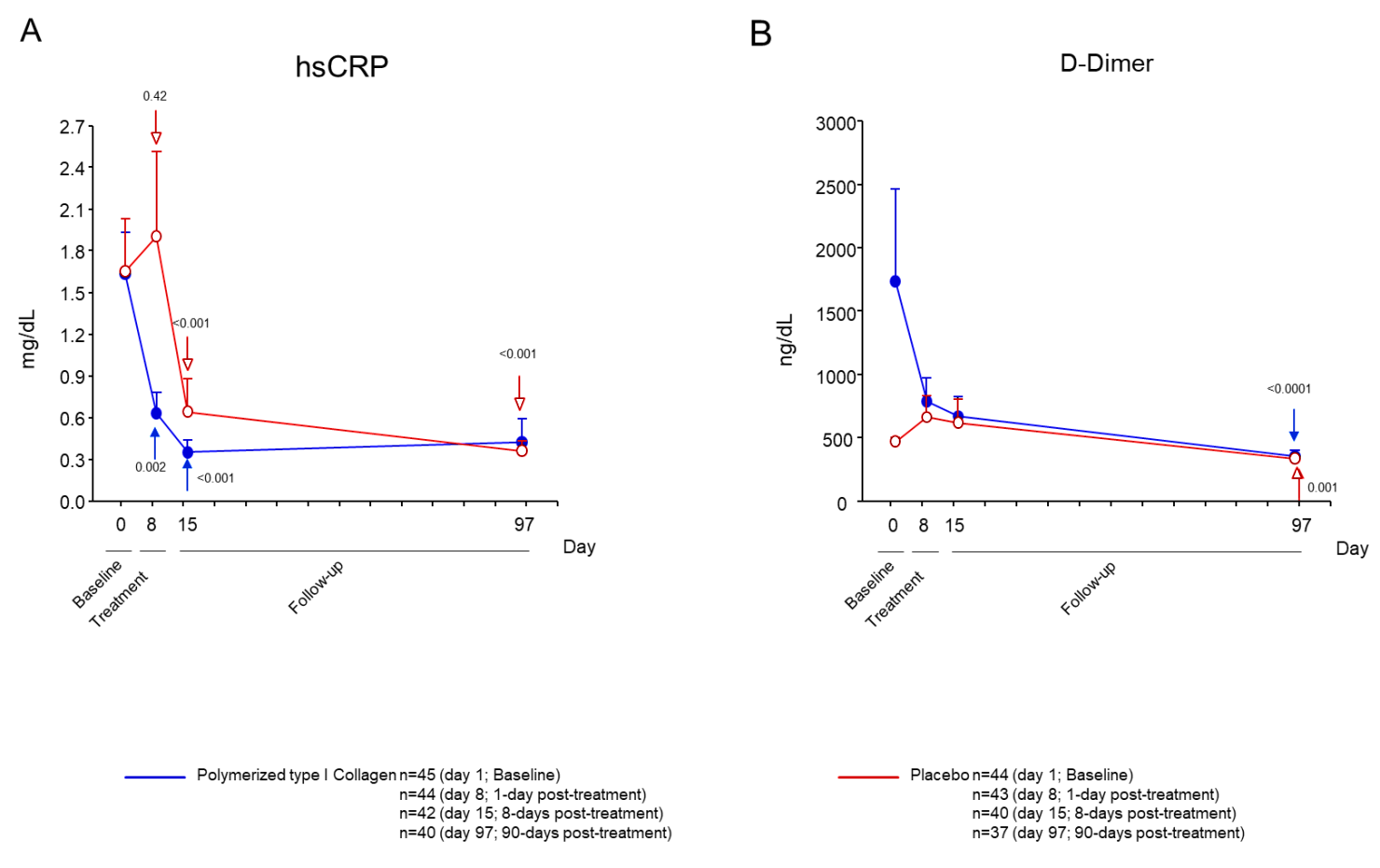
**
